# The health profile of football/soccer players in Northern Ireland – a review of the uefa pre-participation medical screening procedure

**DOI:** 10.1186/2052-1847-6-5

**Published:** 2014-02-13

**Authors:** Neil Heron, ME Cupples

**Affiliations:** 1 Department of General Practice and Primary Care, Queen’s University, Belfast, Irelan; 2 Centre for Public Health Research, Queen’s University, Belfast, Irelan; 3 Centre of Excellence for Public Health Research (NI), Belfast, Irelan

**Keywords:** Pre-participation medical screening, Sudden cardiac death, Football, Soccer, UEFA, Prevention, Family doctors, General practitioners (GPs)

## Abstract

**Background:**

It is compulsory that domestic football/soccer teams in UEFA competitions organise players’ pre-participation medicals. Although screening guidelines have been established, these remain controversial. The findings of medical examinations can have lasting consequences for athletes and doctors. No previous studies have reported UEFA pre-participation screening results in semi-professional footballers. This study aims to further knowledge regarding ‘normal’ data in this population.

**Method:**

Retrospective audit and analysis of records of pre-season medicals for all male first-team players at one semi-professional Northern Ireland Premiership team between 2009-2012. Medicals were conducted by the club doctor following the UEFA proforma. Height, weight, blood pressure (BP), full blood count (FBC), dipstick urinalysis and resting electrocardiogram (ECG) were conducted by an independent nurse. Only one ECG must be documented during a player’s career; other tests are repeated yearly.

**Results:**

89 medicals from 47 players (6 goalkeepers, 11 defenders, 22 midfielders and 8 attackers; mean age 25.0 years (SD 4.86)) were reviewed. Mean height of the players was 179.3 cm (SD 5.90) with a mean weight of 77.6 kg (SD 10.5). Of 89 urine dipsticks, 7 were positive for protein; all 7 were normal on repeat testing following 48 hours of rest. Of 40 ECGs (mean ventricular rate 61.2 bpm (SD 11.6)), one was referred to cardiology (right bundle branch block; prolonged Q-T interval). No players were excluded from participation.

**Conclusions:**

This study provides important information about ‘normal’ values in a population of semi-professional footballers. Urinalysis showing protein is not uncommon but is likely to be normal on repeat testing.

## Background

UEFA (Union of European Football Associations) [1] now endorse yearly medicals for domestic football/soccer teams playing in European competition, in keeping with other sporting governing bodies [2-4]. Pre-participation medicals are therefore now a yearly occurrence for professional footballers and pre-participation screening medicals are also performed prior to footballers signing for new clubs. The medical proforma for professional footballers was first implemented by FIFA (Fédération Internationale de Football Association) [5].

Pre-participation medicals became popular after the findings of the ‘Italian experience’ , with a medical history, physical exam and ECG (electrocardiogram) now mandatory for all Italian sport participants [6]. The main reason for performing the pre-participation medicals is to screen for preventable causes of sudden cardiac death, which has gained much publicity within Europe recently with the collapse and successful resuscitation of Fabrice Muamba when playing for Bolton FC in 2012 [7]. As well as identifying potential preventable causes of sudden cardiac death in athletes, pre-participation medical screening is proposed as a time to identify any potential medical issues and optimise treatment. Family doctors (GPs) are often asked to perform these medicals either through direct employment with clubs or during their day-to-day work in the community.

Previous publications have focused on specific issues found within pre-participation medicals, for example hip strength asymmetry [8] or blood pressure (BP) [9]; a few studies have presented findings from pre-competition medicals in elite footballers [8-11]. However there is a lack of published literature regarding the pre-participation medical screening results for semi-professional footballers, particularly within the context of the UEFA pre-participation medical.

A diagnostic dilemma for the sports physician conducting these medicals is the question of what is ‘normal’? For example, it may be difficult to differentiate between physiological changes in the heart as part of its response to exercise (the ‘athletes’ heart’) and hypertrophic obstructive cardiomyopathy (HOCM) [12]. Further studies are therefore required to provide sport physicians with information to help them identify when further investigation is warranted and to allow scientists to profile the characteristics of successful footballers, which may inform the selection or management of the next generation of professional players [13]. The aim of this study is to audit the findings from four years of pre-season medicals performed according to the UEFA medical requirements, in a semi-professional football team playing in the Northern Ireland Premier League from seasons 2008/9 to 2012/3. No ethical approval was required for this audit.

## Methods

Between June 2008 and June 2012 across four seasons, all 47 male first-team players from one semi-professional football side underwent pre-participation medical screening examinations as per the UEFA medical guidelines for clubs participating in European competition (Additional file 1). All players were white and from Northern Ireland descent. The pre-participation medical screening included a medical history, physical examination, full blood count and urinalysis undertaken annually for every first team player as per the UEFA medical proforma. Players were asked how many games they played in the previous season, including first team, reserve and friendly matches. At least one ECG was recorded for each player aged over eighteen years. The club doctor who performed the medicals was a family doctor (member of the Royal College of General Practitioners, MRCGP) with specialist training in sports medicine (member of the Faculty of Sport and Exercise Medicine, MFSEM UK). Athletes with positive findings were referred as appropriate for a second opinion. Consent was obtained from all athletes for analysis and reporting of their anonymised data.

The medical was generally carried out in June of each year, prior to the start of the season. The resting ECG, urinalysis, height, weight and full blood count (FBC) were conducted by an independent, cardiac technician. BP and resting heart rate were recorded after five minutes of rest in a sitting position [14], using a validated and calibrated electronic BP monitor (Omron M5-I). Systolic and diastolic BP were recorded as the mean of two consecutive BP recordings [14]. Hypertension was defined as systolic BP equal to or greater than 140 mmHg and/or diastolic BP equal to or greater than 90 mmHg [15]. Weight and height were measured and body mass index calculated as weight in kilograms divided by height in metres squared. Height was measured without shoes to the nearest half a centimeter using a calibrated stadiometer (SECA Leicester Height Measure). Body weight was measured in light clothing and without shoes to the nearest 0.1 kg using a standard physician’s balance scale (manufacturer Tanita).

A random, fresh urine sample was tested for glucose and protein using Multistix test strips (Siemens Medical Solutions Diagnostics) within 1 hour of being produced. Five millilitres of blood was obtained for a full blood count (FBC), analysed on the same day it was produced in an accredited laboratory.

### Electrocardiography

Standard 12-lead ECGs (manufacturer GE) were performed with the subject in a supine position [16] and were read by the club doctor (NH). Only one ECG has to be reported for any player during his professional career and an ECG is therefore not carried out on every player every year. Ventricular rate, PR interval, QRS duration, and QT interval were measured.

### SOLEC (Standing on one leg with eyes closed) test

The SOLEC test, optional within the UEFA medical pre-participation screening proforma, is performed by timing how long a player can stand on one leg with eyes closed. The best time after three trials is recorded.

### Statistical analysis

Statistical analysis was performed using SPSS v 19, and Microsoft Excel 2007. Frequencies, cross-tabulations, means and standard deviations are reported.

## Results

### Demographics

There were 89 pre-participation medical screenings conducted in 47 players (Table 1), including 6 goalkeepers; 11 defenders; 22 midfielders; 8 attackers. Their mean age was 25.0 years (SD 4.86). The players had played an average of 38 games in the season previous to their medical; 24 players reported that their dominant foot was left, with no players reporting dual dominance.

**Table 1:** Player Demographics

**Player measurement**	**Mean (Standard deviation)**
Age (years)	25.0 (4.9)
Height (cms)	179.3 (5.9)
Weight (kg)	77.6 (10.5)
BMI (kg/m^2^)	24.1 (2.5)
Systolic blood pressure (mmHg)	122.5 (7.1)
Diastolic blood pressure (mmHg)	77.7 (6.9)
Games played in previous season	38 (11.3)

### Medical history

Overall, 37 players reported a family history of medical conditions in a first degree relative: diabetes mellitus was the most commonly reported (Table 2); 14 players reported a past medical history of asthma (Table 3). Regular medication was reported in 16 medicals; 9 reported use of a salbutamol inhaler, 4 a corticosteroid inhaler, 2 insulin and use of each of neocarbimazole, flucloxacillin, roacutane, diclofenac and epilim was reported once.

**Table 2:** Family history of medical complaints

**Medical condition reported in a first-degree relative**	**Number of times reported in the pre-participation medical screening (n-89)**
Type two diabetes mellitus	12
Type one diabetes mellitus	10
Ischaemic heart disease	10
Hypertension	10
Cerebrovascular disease	3
Hypothyroidism	2
Lupus	2
Atrial fibrillation	2
Bowel cancer	1
Rheumatoid arthritis	1

**Table 3:** Personal history of medical complaints

**Medical condition reported in past medical history**	**Number of times reported by a player during the pre-participation medicals (n-89)**
Asthma	14
Tibia/fibula fracture	5
Lateral and medial ligament of knee strain (grade 1, 2, 3)	4
Metatarsal fracture	4
Patella tendinitis	3
Right clavicular fracture	2
Meniscectomy	2
Type one diabetes mellitus	2
Rotator cuff tear	2
Metacarpal fracture	2
Celiac disease	2
ACL cruciate rupture	2
Epilepsy	2
*Other	20

*Other – includes one report each of toe dislocation (distal interphalangeal joint), cerebral abscess, knee osteoarthritis, posterior cruciate ligament rupture, pilonidal sinus, ankle lateral ligament sprain ( grade 1, 2, or 3), post-concussive syndrome, scaphoid fracture, acne, hyperthyroid, pes planus (flat feet), hamstring strain (grade 1, 2 or 3), left testicular cyst, iliotibial band syndrome, fractured nose, fractured mandible, gastrocnemius strain (grade 1, 2 or 3), osteitis pubis, recurrent shoulder dislocation, and hayfever.

Thirteen players were taking regular supplements; nine reported regular use of protein and amino acid supplements, five used creatinine and a multi-vitamin, four used glucosamine and one cod liver oil.

Two players reported medical issues within the month preceeding screening: one had conjunctivitis treated with oral antibiotics and one was investigated for renal calculi. All players’ routine vaccines, including tetanus, were up-to-date.

### Examination findings

All players had head and neck, lymphatic, respiratory, cardiovascular, abdominal, peripheral vascular, neurological, and motor system examinations. None had any hypertensive BP recordings. One player had acne requiring oral treatment with vitamin A (roacutane), prescribed prior to signing for the club.

Nine players had a positive finding on motor system examination. These included reduced internal rotation of both hips; Ober test positive, with a history of iliotibial band syndrome (n = 1); knee crepitus (n = 3); tight adductors; and tight hamstrings bilaterally (n = 3), with less than 45 degrees on straight leg raising.

### Urine dipstick analysis

No urine dipstick analysis tested positive for glucose. Seven players tested positive for protein. On all occasions this occurred after prolonged standing due to the nature of the player’s day job (e.g. factory work) or after an intense episode of exercise. All re-test urine dipstick analyses were negative following 48 hours of rest and adequate hydration.

### SOLEC test

The SOLEC test, performed on 47 players, yielded a mean score for the right leg of 29.2 seconds (SD 16.0), and for the left leg 31.5 seconds (SD 14.1).

### Full blood count

Two FBCs were conducted by the players’ own general practitioner and these results are not available for review. Of 87 FBCs analysed the mean haemoglobulin was 14.68 g/dl (SD 0.79) and the mean haematocrit was 0.447 l/l (standard deviation 0.028). The mean red cell count was 4.82 × 10^12^/l (standard deviation 0.25) with the average mean cell volume 92.87 fL (standard deviation 5.24). The mean platelets were 229.67 × 10^9^/l (standard deviation 46.83) and the average white cell count was 6.95 × 10^9^/l (standard deviation 1.72).

### Electrocardiograms (ECGs)

40 ECGs were conducted, showing a mean resting ventricular rate of 61.2 beats/minute (SD 11.6) (Table 4). One player was referred for cardiology review due to a prolonged QTc at 437 ms associated with right bundle branch block. Of note, his echocardiogram was reported as being within normal limits.

**Table 4:** Players’ ECG variables

**ECG variable**	**Mean**	**Standard deviation**
Ventricular rate (bpm)	61.2	11.6
PR interval (ms)	159.3	33.7
QRS duration (ms)	99.1	10.0
QT (ms)	410.5	27.9
QTc (ms)	410	21.3

### Summary assessment

All 47 players from the 89 medicals were declared as eligible for competitive soccer.

## Discussion

This study reviewed the health profile of soccer players in Northern Ireland (NI) as described through the UEFA pre-participation medical screening procedure. The club at which the medicals were performed is one of the most successful currently in NI, winning four top flight trophies over the period of time for which the medicals were conducted and having three full NI internationals amongst their players.

Pre-participation medical screening of athletes is a controversial topic [17,18]. It does not fulfill the requirements for an appropriate screening test [19], to detect the known causes of sudden cardiac death in athletes [20]. However, the medical allows a time to review the athlete, identify potential injury risk factors [21], help prevent injuries and provide health education, e.g. anti-doping guidelines [22], and maximise recovery strategies for the athlete [23]. There is also a need to optimise treatment of current medical conditions such as asthma [24].

One of the biggest risk factors for injury in elite footballers is a previous history of injury [25,26] and an important part of the pre-participation medical screening is therefore to be aware of all players’ injury history and to instigate preventative work as required. For example the player with pes planus was referred for custom-made insoles, the player with iliotibial band syndrome was started on an appropriate stretching programme and the player with osteitis pubis was advised to report any hip or groin symptoms early to allow appropriate modification of his training load.

It is vital for the team physician to know what medical conditions exist in the team, to determine medications that need to be carried. This study’s findings highlight how medications may be needed to deal with emergencies related to diabetes, asthma or epilepsy and how appropriate therapeutic use exemptions (TUEs) may be requested.

The study data reflect use of sport supplementation, which is becoming more common amongst footballers [27]. Sport physicians need to provide appropriate education to players regarding supplement use and the issue of contamination [28]. One area which the club could improve upon is the provision of a nutritionist to provide dietary advice.

Testing for urinary glucose is a poor screening test for diabetes [29]. Dipstick urinalysis for proteinuria has been reported to be a poor screening test for renal damage, particularly within pre-participation medical screening examinations [30]. Physiological reasons for proteinuria amongst athletes include exercise, particularly of an intense nature [30], and prolonged standing. The club now reinforces with players the need for relative rest 48 hours prior to a medical and advises adequate hydration to avoid false positives for proteinuria.

Previous authors have commented that tinea pedis is common amongst athletes [31]. The low prevalence of this fungal infection in our cohort may be explained by the emphasis which the club places on foot hygiene, for example wearing flip-flops in the showering area.

The players with evidence of knee crepitus and reduced internal rotation at the hip may be developing early signs of osteoarthritis of these joints and sporting participation, with or without joint injury, appears to be a risk factor for early development of osteoarthritis [32]. Athletes therefore need to be counseled appropriately regarding future elite sporting participation and may need to consider early retirement from professional sport.

All the players in our cohort were found to be normotensive. One previous study reviewing BP in Norwegian footballers using ambulatory BP monitors, found that 32% of their cohort had masked hypertension [14]. The use of ambulatory BP monitoring instead of office BP recordings may be of interest although the cost may be prohibitive.

Poor ankle stability as measured by the SOLEC test is reported to increase the risk of injury [33], particularly of the ankle and knee regions [34], with proprioception training in footballers with previous ankle inversion injuries reducing further ankle sprains [35]. The players averaged approximately 30 seconds on each leg. Previous authors have advised that if players are unable to undertake the SOLEC test for at least 60 seconds on each leg, then balance training using a balance board should be instigated, with positive results following 3 months of training [33]. As a result proprioception work has now been integrated routinely into the club’s training sessions.

The height, weight and body mass index (BMI) of the semi-professionals in our cohort were consistent with a previous study looking at professional footballers in the top four European leagues [36]. Although despite being of a similar average height, our players were nearly 8 kg heavier than a cohort of footballers from the Czech Republic which included amateur and professional players [37]. A measurement which may be of more relevance is percentage body fat [38].

Typical findings reported by previous authors reviewing athletic ECGs have included sinus bradycardia and first-degree AV block with a PR interval of greater than 200 ms [39]. The average ventricular rate of our cohort was 61 bpm with a PR interval of 159.3 ms. Athletic changes to the heart may occur after a minimum of four hours activity per week [39]: our semi-professional cohort might only train for approximately three hours a week. Any abnormal ECG findings would require further investigation with an echocardiogram, as suggested by previous authors [17].

There is some evidence to suggest that as age increases, the risk of hamstring strains increases [40,41] and indeed the rate of all muscular injuries. A common injury within football/soccer is groin injuries [42-44]. Dallinga et al. [40] report in a systematic review that hip adduction-to-abduction strength ratio was a significant predictor of a future adductor strain (RR-17, based on a hip adduction of <80% of abduction strength), which is supported by previous authors [43,45]. Heavier and shorter players are also reported to be at increased risk of quadriceps strains [46,47]. During the pre-participation medical screening players need to be educated about these facts with attempts made to modify the other intrinsic risk factors, e.g. flexibility and strength deficits [47], to allow them to reduce their risk from this injury.

Full blood count parameters in elite German footballers have been reported [48] but this is the first time that such parameters have been documented in NI footballers. As would be expected in an athletic cohort, the mean values for haematocrit and haemoglobulin are at the high end of the normal range for the general population.

### Limitations

This study is only of one team, reporting on a relatively small cohort and over the course of four seasons. One un-blinded doctor conducted the medicals, therefore ensuring a consistent approach. However, possible bias in observations may exist. Whilst the study reviews medicals performed before the start of the season, further relevant detail may be elicited if middle and end of the season medicals were conducted. The preseason medical largely relies on player recall of data, e.g. previous injuries, which may underestimate the prevalence of certain conditions. Further details of ECG changes (ST and T wave changes, etc) may have been identified if the ECGs were read by independent, blinded cardiologists.

## Conclusions

A standardised pre-participation medical is feasible within European football and can be performed within small member nations of UEFA, such as Northern Ireland. Of the 89 medicals presented here, ten players had a positive finding on physical examination, seven on initial urinalysis, and one positive finding on the resting ECG. Positive urine protein dipstick results need to be confirmed on a second test following 48 hours of rest and adequate hydration. Only one player required onward referral to a cardiologist for suspicious findings on a resting ECG.

This study has therefore established ‘normal’ findings for sport physicians to refer to when conducting pre-participation medicals in the football athletic population, particularly within semi-professional footballers. To facilitate further knowledge translation, comparison of the pre-participation medical findings with subsequent findings or injuries later in the season should help to optimize preventive interventions and minimize the risk of injury or poor health for all footballers playing in the top flight of the game.

### What this study adds

• An audit of pre-participation medicals as per the UEFA guidelines conducted in a population of Northern Ireland semi-professional footballers for the first time.

• This audit helps to establish ‘normal’ values for other sport physicians conducting pre-participation medicals in semi-professional and Northern Irish athletes to refer to.

• Urine protein dipstick urinalysis can be positive following exercise or prolonged standing and it is therefore important to conduct this test after 48 hours of relative rest and appropriate hydration to avoid false-positive results.

• Positive ECG findings in athletes can be further investigated by echocardiography and, if required, appropriate onward referral to cardiology.

## Competing interests

The author’s declare that they have no competing interests.

## Authors’ contributions

NH conceived the study. NH carried out the study, performed the statistical analysis, and drafted the manuscript. All authors participated in the design of the study, with MEC providing supervision throughout the study, helping with study analysis and reviewing successive drafts of the manuscript. All authors read and approved the final manuscript.

## Pre-publication history

The pre-publication history for this paper can be accessed here:

http://www.biomedcentral.com/2052-1847/6/5/prepub

## Supplementary Material

Additional file 1 **UEFA Pre-Participation Medical Screening Proforma.** Medical Care of Players. Click here for file

## References

[B1] UEFA Regulations of the UEFA Europa League 2011/12 UEFA regulations 2011 1 1 1

[B2] Corrado D Pelliccia A Bjornstad H Vanhees L Biffi A Borjesson M UEFA Cardiovascular pre-participation screening of young competitive athletes for prevention of sudden death: proposal for a common European protocol - Consensus statement of the Study Group of Sport Cardiology of the Working Group of Cardiac Rehabilitation and Exercise Physiology and the Working Group of Myocardial and Pericardial Diseases of the European Society of Cardiology Eur Heart J 2005 26 5 516 524 15689345 10.1093/eurheartj/ehi108

[B3] Maron B Thompson P Ackerman M Balady G Berger S Cohen D Dimeff R Douglas PS Glover DW Hutter AM Jr Krauss MD Maron MS Mitten MJ Roberts WO Puffer JC Recommendations and considerations related to preparticipation screening for cardiovascular abnormalities in competitive athletes: 2007 update: a scientific statement from the American Heart Association Council on Nutrition, Physical Activity, and Metabolism: endorsed by the American College of Cardiology Foundation Circulation 2007 115 2 1643 455 17353433 10.1161/CIRCULATIONAHA.107.181423

[B4] Ljungqvist A Jenoure P Engebretsen L Manuel Alonso J Bahr R Clough A De Bondt G Dvorak J Maloley R Matheson G Meeuwisse W Meijboom E Mountjoy M Pelliccia A Schwellnus M Sprumont D Schamasch P Gauthier JB Dubi C Stupp H Thill C The International Olympic Committee (IOC) Consensus Statement on periodic health evaluation of elite athletes March 2009 Br J Sports Med 2009 43 9 631 643 10.1136/bjsm.2009.064394 19734496

[B5] Dvorak J Grimm K Schmied C Junge A Development and implementation of a standardized precompetition medical assessment of international elite football players–2006 FIFA World Cup Germany Clin J Sport Med 2009 19 4 316 321 10.1097/JSM.0b013e3181b21b6e 19638827

[B6] Corrado D Basso C Pavei A Michieli P Schiavon M Thiene G Trends in sudden cardiovascular death in young competitive athletes after implementation of a preparticipation screening program JAMA-J American Med Association 2006 296 13 1593 1601 10.1001/jama.296.13.1593 17018804

[B7] Fabrice M 2012 fabrice-muamba.com. 17th March 2012. http://fabrice-muamba.com/17-march-2012/ 2012

[B8] Thorborg K Couppé C Petersen J Magnusson S Hölmich P Eccentric hip adduction and abduction strength in elite soccer players and matched controls: a cross-sectional study Bri J Sport Med 2011 45 1 10 13 10.1136/bjsm.2009.061762 19850576

[B9] Berge H Gjerdalen G Andersen T Solberg E Steine K Blood pressure in professional male football players in Norway J Hypertens 2013 Epub 10.1097/HJH.0b013e32835eb5fe 23442990

[B10] Dvorak J Grimm K Schmied C Junge A Feasibility of precompetition medical assessment at FIFA World Cups for female youth players Bri J Sport Med 2012 46 16 1132 1133 10.1136/bjsports-2011-090374 PMC3596861 22021353

[B11] Kervio G Pelliccia A Nagashima J Wilson M Gauthier J Murayama M Uzan L Ville N Carre F Alterations in echocardiographic and electrocardiographic features in Japanese professional soccer players: comparison to African-Caucasian ethnicities Eur J Prev Cardiol 2012 Epub 10.1177/2047487312447905 22548966

[B12] Pelliccia A Di Paolo F Maron B The athlete’s heart: remodeling, electrocardiogram and preparticipation screening Cardiol Rev 2002 10 2 85 90 10.1097/00045415-200203000-00006 11895574

[B13] Williams A Reilly T Talent identification and development in soccer Can J Sport Sci 2000 18 9 657 667 10.1080/02640410050120041 11043892

[B14] Berge HM Andersen TE Solberg EE Steine K High ambulatory blood pressure in male professional football players Br J Sports Med 2013 47 8 521 525 10.1136/bjsports-2013-092354 23501835

[B15] Mancia G Laurent S Agabiti-Rosei E Ambrosioni E Burnier M Caulfield M Reappraisal of European guidelines on hypertension management: a European Society of Hypertension Task Force document J Hypertens 2009 27 11 2121 2158 10.1097/HJH.0b013e328333146d 19838131

[B16] Papadakis M Carre F Kervio G Rawlins J Panoulas VF Chandra N Basavarajaiah S Carby L Fonseca T Sharma S The prevalence, distribution, and clinical outcomes of electrocardiographic repolarization patterns in male athletes of African/Afro-Caribbean origin Eur Heart J 2011 32 18 2304 2313 10.1093/eurheartj/ehr140 21613263

[B17] Pigozzi F Spataro A Fagnani F Maffulli N Preparticipation screening for the detection of cardiovascular abnormalities that may cause sudden death in competitive athletes British J Sports Med 2003 37 1 4 5 10.1136/bjsm.37.1.4 PMC1724593 12547737

[B18] Asif I Rao A Drezner J Sudden cardiac death in young athletes: what is the role of screening? Curr Opin Cardiol 2013 28 1 55 62 10.1097/HCO.0b013e32835b0ab9 23196775

[B19] Wilson JMG Jungner G Principles and practise of screening for disease 1964 World Health Organisation (WHO): WHO Public Health Papers 34

[B20] MacAuley D Does preseason screening for cardiac disease really work?: the British perspective Med Sci Sports Exerc 1998 30 Suppl 10 S345 S350 9789860 10.1097/00005768-199810001-00002

[B21] Volpi P Taioli E The health profile of professional soccer players: future opportunities for injury prevention J Strength Cond Res 2012 26 12 3473 3479 10.1519/JSC.0b013e31824e195f 22344052

[B22] McKeag D Preseason physical examination for the prevention of sports injuries Sports Med 1985 2 6 413 431 10.2165/00007256-198502060-00003 3906829

[B23] Higgins T Cameron M Climstein M Evaluation of passive recovery, cold water immersion, and contrast baths for recovery, as measured by game performances markers, between two simulated games of rugby union J Strength Cond Res 2012 Epub 10.1519/JSC.0b013e31825c32b9 22692113

[B24] Ross R The prevalence of reversible airway obstruction in professional football players Med Sci Sports Exerc 2000 32 12 1985 1989 10.1097/00005768-200012000-00003 11128840

[B25] Hägglund M Waldén M Ekstrand J Previous injury as a risk factor for injury in elite football: a prospective study over two consecutive seasons Br J Sport Med 2006 40 9 767 772 10.1136/bjsm.2006.026609 PMC2564391 16855067

[B26] Hägglund M Waldén M Ekstrand J Risk factors for lower extremity muscle injury in professional soccer: the UEFA Injury Study Am J Sports Med 2013 41 2 327 335 10.1177/0363546512470634 23263293

[B27] Taioli E Use of permitted drugs in Italian professional soccer players Br J Sport Med 2007 41 7 439 441 10.1136/bjsm.2006.034405 PMC2465348 17317759

[B28] Judkins C Prock P Supplements and inadvertent doping - how big is the risk to athletes Med Sport Sci 2012 59 143 152 23075565 10.1159/000341970

[B29] James GP Bee DE Glucosuria: accuracy and precision of laboratory diagnosis by dip stick analysis Clin Chem 1979 25 6 996 1001 445837

[B30] Saeed F Naga Pavan Kumar Devaki P Mahendrakar L Holley J Exercise-induced proteinuria? J Fam Pract 2012 61 1 23 26 22220292

[B31] Pickup T Adams B Prevalence of tinea pedis in professional and college soccer players versus non-athletes Clin J Sport Med 2007 17 1 52 54 10.1097/JSM.0b013e31802ed88e 17304007

[B32] Kuijt M Inklaar H Gouttebarge V Frings-Dresen M Knee and ankle osteoarthritis in former elite soccer players: a systematic review of the recent literature J Sci Med Sport 2012 15 6 480 487 10.1016/j.jsams.2012.02.008 22572082

[B33] Ekstrand J Karlsson J Hodson A Football Medicine 2003 104 105

[B34] Alentorn-Geli E Myer GD Silvers HJ Samitier G Romero D Lázaro-Haro C Cugat R Prevention of non-contact anterior cruciate ligament injuries in soccer players. Part 1: Mechanisms of injury and underlying risk factors. Knee Surgery, Sports Traumatology, Arthroscopy Official J ESSKA 2009 17 7 705 729 10.1007/s00167-009-0813-1 19452139

[B35] Mohammadi F Comparison of 3 preventive methods to reduce the recurrence of ankle inversion sprains in male soccer players Am J Sports Med 2007 35 6 922 926 10.1177/0363546507299259 17379918

[B36] Bloomfield J Polman R Butterly R O’Donoghue P Analysis of age, stature, body mass, BMI and quality of elite soccer players from 4 European Leagues J Sports Med Phys Fitness 2005 45 1 58 67 16208292

[B37] Junge A Dvorak J Chomiak J Peterson L Graf-Baumann T Medical History and Physical Findings in Football Players of Different Ages and Skill Levels Am J Sports Med 2000 28 suppl 5) S-16 S-21 11032103

[B38] Etchison WC Bloodgood EA Minton CP Thompson NJ Collins MA Hunter SC Dai H Body mass index and percentage of body fat as indicators for obesity in an adolescent athletic population Sports Health 2011 3 3 249 252 10.1177/1941738111404655 23016014 PMC3445161

[B39] Drezner JA Fischbach P Froelicher V Marek J Pelliccia A Prutkin JM Sharma S Wilson MG Ackerman MJ Anderson J Ashley E Asplund CA Baggish AL Börjesson M Cannon BC Corrado D DiFiori JP Harmon KG Heidbuchel H Owens DS Paul S Salerno JC Stein R Vetter VL Normal electrocardiographic findings: recognising physiological adaptations in athletes Br J Sport Med 2013 47 3 125 136 10.1136/bjsports-2012-092068 23303759

[B40] Dallinga J Benjaminse A Lemmink K Which screening tools can predict injury to the lower extremities in team sports?: a systematic review Sports Med 2012 42 9 791 815 10.1007/BF03262295 22909185

[B41] Freckleton G Pizzari T Risk factors for hamstring muscle strain injury in sport: a systematic review and meta-analysis Br J Sport Med 2013 47 351 358 10.1136/bjsports-2011-090664 22763118

[B42] Paajanen H Ristolainen L Turunen H Kujala U Prevalence and etiological factors of sport-related groin injuries in top-level soccer compared to non-contact sports Arch Orthop Trauma Surg 2011 131 2 261 266 10.1007/s00402-010-1169-1 20714902

[B43] Engebretsen A Myklebust G Holme I Engebretsen L Bahr R Intrinsic risk factors for groin injuries among male soccer players: a prospective cohort study Am J Sports Med 2010 38 10 2051 2057 10.1177/0363546510375544 20699426

[B44] Hölmich P Larsen K Krogsgaard K Gluud C Exercise program for prevention of groin pain in football players: a cluster-randomized trial Scand j med scie sports 2010 20 6 814 821 10.1111/j.1600-0838.2009.00998.x 19883386

[B45] Jensen J Hölmich P Bandholm T Zebis M Andersen L Thorborg K Eccentric strengthening effect of hip-adductor training with elastic bands in soccer players: a randomised controlled trial Br J Sports Med 2012 Epub 10.1136/bjsports-2012-091095 22763117

[B46] Fousekis K Tsepis E Poulmedis P Athanasopoulos S Vagenas G Intrinsic risk factors of non-contact quadriceps and hamstring strains in soccer: a prospective study of 100 professional players Br J Sports Med 2011 45 9 709 714 10.1136/bjsm.2010.077560 21119022

[B47] Mendiguchia J Alentorn-Geli E Idoate F Myer G Rectus femoris muscle injuries in football: a clinically relevant review of mechanisms of injury, risk factors and preventive strategies Br J Sports Med 2013 47 359 366 10.1136/bjsports-2012-091250 22864009

[B48] Meyer T Meister S Routine blood parameters in elite soccer players Int J Sports Med 2011 32 11 875 881 22020850 10.1055/s-0031-1280776

