# Supplementary material for: The health profile of football/soccer players in Northern Ireland – a review of the uefa pre-participation medical screening procedure
Source: BMC Sports Sci Med Rehabil. 2014 Feb 13;6:5. doi: 10.1186/2052-1847-6-5 (PMC4021641; doi:10.1186/2052-1847-6-5)
Supplement: Additional file 1 — UEFA Pre-Participation Medical Screening Proforma. Medical Care of Players. [file 2052-1847-6-5-S1.doc]

| **UEFA CLUB LICENSING STANDARD**  ***Medical Care of Players*** | 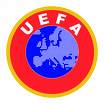 |
| --- | --- |

***Scope***

**Refers to the senior team players that have been listed on a club’s official team line for any first team match during the domestic 2009/10 season up to 31 January 2010.**

***Medical Records***

**The club doctor, or other nominated doctor (approved in advance by the IFA), shall retain up to date individual medical records for each player. This file shall be subject to medical confidentiality and shall contain the results and reports of previously performed medical examinations.**

***Medical Examinations***

**Each player within the scope shall be subject to medical examinations and tests to be carried out on an annual basis (with the exception of ECG and Echo tests) detailed below.**

***Special Cardiological Examination***

**According to the criterion, a “cardiovascular screening” must be part of the yearly medical examination. However, after consultation of the UEFA Medical Committee it has been defined that for the purpose of club licensing, every first squad player must undergo a yearly medical examination (minimum content see below), but a cardiological examination (see D below) does not need to be performed every year to every first squad player.**

**Instead, it has been defined that every player who belongs to the first squad of a club must have in his personal medical records, as a minimum, one electrocardiogram and one echocardiography. For youth players being part of the first squad, these two cardiological examinations must be performed at the latest before their 21^st^ birthday (see D below).**

**With the exception of the cardiological (see D below) and laboratory examinations (See E below), those checks defined as mandatory may be performed without any special equipment and should not cause high additional costs.**

**In addition to the mandatory minimum medical checks, details below include optional examinations which are considered as best practice recommendations. Based on the results of the medical examinations and upon the professional judgement of the medical doctor, subsequent checks may be indicated to ensure an adequate medical follow-up of the player. However, such additional checks go beyond the scope of this criterion.**

**MEDICAL QUESTIONNAIRE – *SUMMARY PAGE***

| **Player Name** |  | **Date of Birth** |  |
| --- | --- | --- | --- |
| **Current Club** |  | **Date of Examination** |  |

| **Mandatory Medical Examinations/Tests** | **Notes** |
| --- | --- |
| **Personal Football History** |  |
| **Medical History** |  |
| **General Medical Exam** |  |
| **Electrocardiogram**  **Echocardiography** |  |
| **Lab Exam (Blood)**  **Lab Exam (Urine)** |  |
| **Orthopaedic** |  |

I can confirm that the medical records for the player,______________________, are up to date and he has / has not* passed all of the medical examinations as stated above. *(*Delete as appropriate)*

| **DOCTOR** | **CLUB CHAIRMAN** |
| --- | --- |
| **Name:** | **Name:** |
| **Signature:** | **Signature:** |
| **Date:** | **Date:** |

| **A) Personal football history** | |
| --- | --- |
| The personal football history represents the football-specific basis for the medical examination. It should be documented and kept up-to-date throughout the player’s career.  UEFA recommends these recordings as best practice following several football-specific medical research studies that would assist medical doctors with their internal medical audit. | |
| 1. **Total number of matches played in previous season** (incl. friendly matches) | **recommended annually** |
| 1. **Dominant leg** |
| 1. **Position on the field** |

| **B) Medical history and heredity of the player** | |
| --- | --- |
| This general part ‘Medical history and heredity’ is the starting point for the player’s medical record. It is essential that the outcome of these checks is kept up-to-date throughout the player’s career. | |
| 1. **Family history (1^st^ generation, i.e. parents, brothers and sisters)** 2. Hypertension, stroke; 3. Heart conditions incl. sudden cardiac death; 4. Vascular problems, varicose, deep venous thrombosis; 5. Diabetes; 6. Allergies, asthma; 7. Cancer, blood disease; 8. Chronic joint or muscle problems; 9. Hormonal problems. | **mandatory, to be updated annually** |
| 1. **Medical history of the player** 2. Heart problems, arrhythmias, syncope; 3. Concussion; 4. Allergies, asthma; 5. Recurrent infections; 6. Major diseases; 7. Major injuries causing surgery, hospitalisation, absence from football of more than 1 month. | **mandatory, to be updated annually** |
| 1. **Present complaints** 2. Symptoms such as pain in general (muscle, articulation); 3. Chest pain, dyspnoea, palpitation, arrhythmia; 4. Dizziness, syncope; 5. Flu-like symptoms, cough, expectoration; 6. Loss of appetite, weight loss; 7. Sleeplessness; 8. Gastrointestinal upset. | **mandatory annually** |
| 1. **Medication / supplements** 2. Current specific medication being taken by the player; 3. Evidence that a TUE (Therapeutic Use Exemption) has been granted (if required); 4. Nutritional supplements being taken by the player; 5. Player educated about Anti-Doping Codes. | **mandatory annually** |
| 1. **Vaccination**   Record of status of vaccination (incl. date);  Strongly recommended:  Vaccination against Tetanus and Hepatitis A and B | **mandatory, to be updated annually** |

| **C) General medical examination** | |
| --- | --- |
| This is the 2^nd^ part of the doctor’s routine physical examination. | |
| 1. **Height** | **mandatory annually** |
| 1. **Weight** |
| 1. **Blood pressure** (to ensure validity of continuous testing, it is recommended to always use the same arm and to specify it in the player’s medical records) |
| 1. **Head and neck (eyes with vision test, nose, ears, teeth, throat, thyroid gland)** |
| 1. **Lymph nodes** |
| 1. **Chest and lungs (inspection, auscultation, percussion, inspiratory and expiratory chest expansion)** |
| 1. **Heart (sounds, murmurs, pulse, arrhythmias)** |
| 1. **Abdomen (incl. hernia, scars)** |
| 1. **Blood vessels** (e.g. peripheral pulses, vascular murmurs, varicoses) |
| 1. **Skin inspection** |
| 1. **Nervous system** (e.g. reflexes, sensory abnormalities) |
| 1. **Motor system** (e.g. weakness, atrophy) |

| **D) Special cardiological examination** | |
| --- | --- |
| As a principle, a standard 12-lead electrocardiogram (ECG) and an echocardiography must be performed at the earliest opportunity during the career of a player and in particular if indicated by clinical examination. If indicated by anamnestic and clinical indication it is recommended to perform repeated testing including an Exercise-ECG and an echocardiography.  For the purpose of club licensing, it is mandatory to perform one standard 12-lead ECG and one echocardiography   1. to all players who belong to the first squad at the latest before their 21^st^ birthday; and 2. to all players who are older than 21 years and belong to the first squad if they have not yet an ECG and echocardiography in their personal medical records.   **The result of the performed examinations must be contained in the player’s medical records.** | |
| 1. **Electrocardiogram (12-leads ECG)** | **mandatory according to I) and II) above** |
| 1. **Echocardiography** |

| **E) Laboratory examination** | |
| --- | --- |
| Clubs involved in UEFA competitions will normally have a multinational squad. Therefore mandatory and strongly recommended tests are detailed below as a means of conducting a comprehensive laboratory screening. This list is by no means complete.  **All laboratory tests must be conducted with the informed consent of the player and be in accordance with national legislation (cf. confidentiality, discrimination issues etc.).** | |
| 1. **Blood count (haemoglobin, haematocrit, erythrocytes, leukocytes, thrombocytes)** | **Mandatory annually** |
| 1. **Urine test** (‘dipstick test’ to determine level of protein and sugar) |
| 1. **Sedimentation rate** | **recommended** |
| 1. **CRP** |
| 1. **Blood fats (cholesterol, HDL- and LDL cholesterol, triglycerides)** |
| 1. **Glucose** |
| 1. **Uric acid** |
| 1. **Creatinine** |
| 1. **Aspartate amino-transferase** |
| 1. **Alanine amino-transferase** |
| 1. **Gamma-glutamyl-transferase** |
| 1. **Creatine kinase** |
| 1. **Potassium** |
| 1. **Sodium** |
| 1. **Magnesium** |
| 1. **Iron** | **recommended** |
| 1. **Ferritin** |
| 1. **Blood group** |
| 1. **HIV test** |
| 1. **Hepatitis screening** |

| **F) Orthopaedic examination and functional tests** | |
| --- | --- |
| The mandatory checks are common in a sports medical examination.  Points 7 to 9 are recommended to assist club doctors with preventive strategies and tests in the rehabilitation of injured players.  In addition, the club doctors are advised to consider the exclusion of the condition of spondylolysis and spondylolisthesis.  References to further assistance in respect of functional tests:   - Simple but reliable functional tests: *Ekstrand J, Karlsson J, Hodson A. Football Medicine. London: Martin Dunitz (Taylor & Francis Group), 2003:562;* - Range of motion and tests for muscle tightness: *Ekstrand J, Wiktorsson M, Öberg B et al. Lower extremity goniometric measurements: a study to determine their reliability. Arch Phys Med Rehabil 1982;63:171-5;* - One-leg hop test: Ageberg E, Zatterstrom R, Moritz U. Stabilometry and one-leg hop test have high test-retest reliability. *Scand J Med Sci Sports 1998;8-4:198-202.* - SOLEC test: *Ageberg E, Zatterstrom R, Moritz U. Stabilometry and one-leg hop test have high test-retest reliability. Scand J Med Sci Sports 1998;8-4:198-202.* | |
| 1. **Spinal column: inspection and functional examination (tenderness, pain, range of movement)** | **mandatory annually** |
| 1. **Shoulder: pain, mobility and stability** |
| 1. **Hip, groin and thigh: pain and mobility** |
| 1. **Knee: pain, mobility, stability and effusion** |
| 1. **Lower leg: pain (shin splint syndrome, achilles tendon)** |
| 1. **Ankle and foot: pain, mobility, stability and effusion** |
| 1. **Range of motion (ROM) and test for muscle tightness**    1. Adductors    2. Hamstrings    3. Iliopsoas    4. Quadriceps    5. Gastrocnemius    6. Soleus | **recommended** |
| 1. **Muscle strength** (one leg hop test) |
| 1. **Muscle balance test** (SOLEC-test: standing one leg eyes closed) |

| **G) Radiological examination and ultrasound scan** |
| --- |
| If indicated by clinical and functional findings out of the medical examination performed, a radiological examination including ultrasound scan, X-ray and MRI may be appropriate.  Performed radiographies, particularly after injuries, must be part of the player’s medical records. |
